# Supplementary material for: Synthetic auxotrophy reveals metabolic regulation of plasma cell generation, affinity maturation, and cytokine receptor signaling
Source: eLife. 2026 Oct 29;14:RP107627. doi: 10.7554/eLife.107627 (PMC13719169; doi:10.7554/eLife.107627)
Supplement: Supplementary file 1 [file elife-107627-fig1-figsupp2-data2.pdf]

GLS

{four independent samples +/+ *versus* four Δ/Δ

actin

M

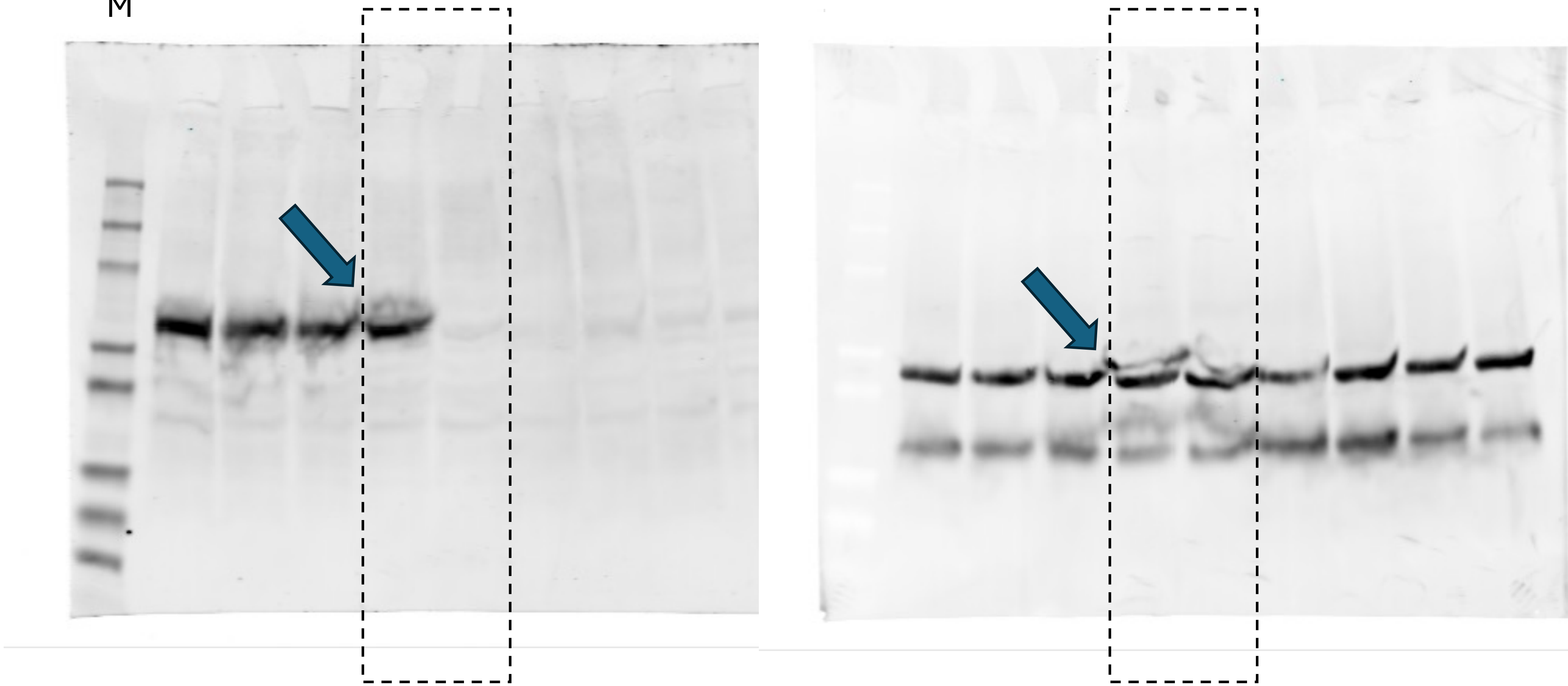

M = size markers
