# Supplementary material for: Synthetic auxotrophy reveals metabolic regulation of plasma cell generation, affinity maturation, and cytokine receptor signaling
Source: eLife. 2026 Oct 29;14:RP107627. doi: 10.7554/eLife.107627 (PMC13719169; doi:10.7554/eLife.107627)
Supplement: Supplementary file 17 [file elife-107627-fig4-figsupp2-data1.zip › source data - Figure 4, supplement 2/P169x2 - Calibration accuracy in QCs.pdf]

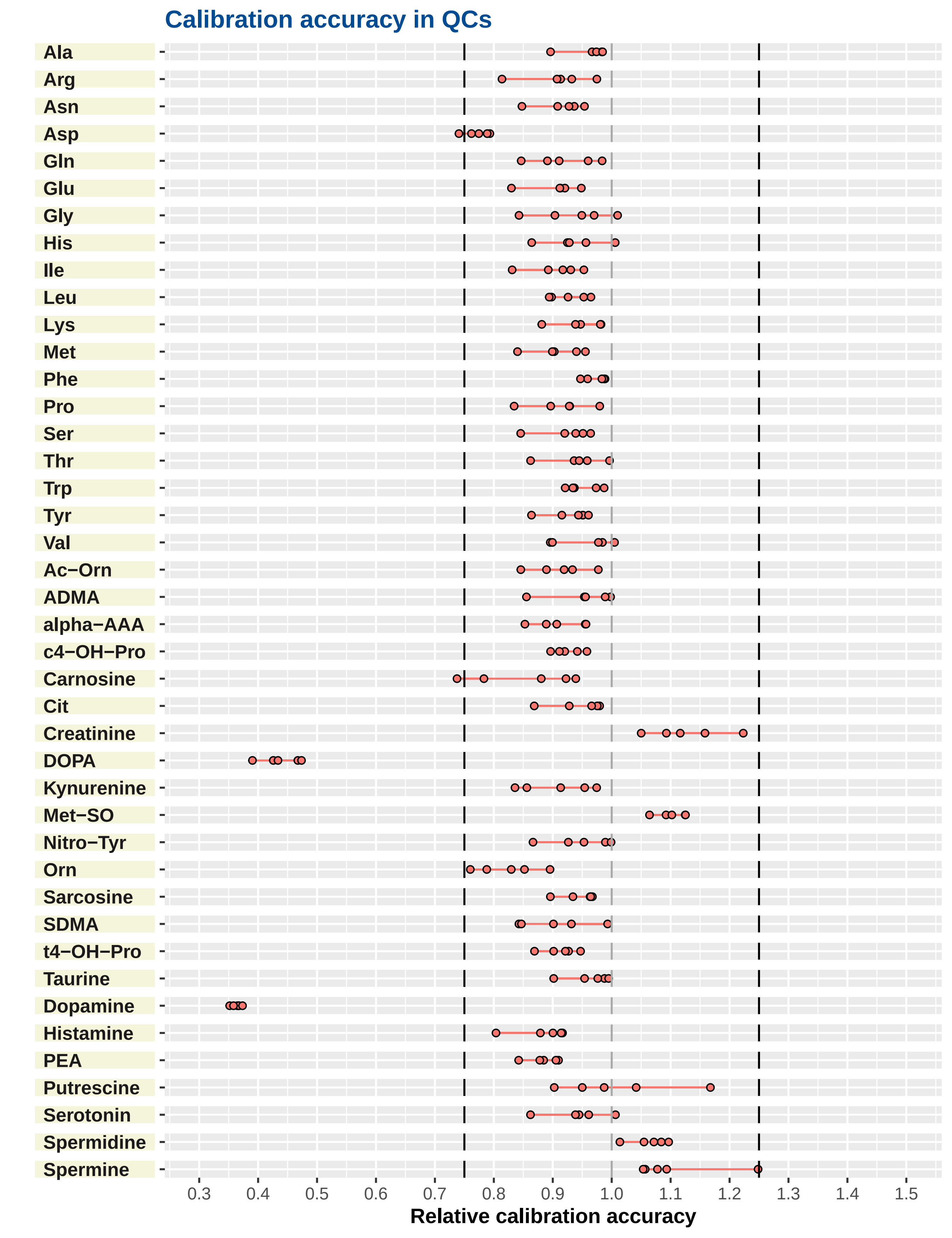

Calibration accuracy in QCs
Ala
Arg
Asn
Asp
Gln
Glu
Gly
His
Ile
Leu
Lys
Met
Phe
Pro
Ser
Thr
Trp
Tyr
Val
Ac−Orn
ADMA
alpha−AAA
c4−OH−Pro
Carnosine
Cit
Creatinine
DOPA
Kynurenine
Met−SO
Nitro−Tyr
Orn
Sarcosine
SDMA
t4−OH−Pro
Taurine
Dopamine
Histamine
PEA
Putrescine
Serotonin
Spermidine
Spermine
0.3
0.4
0.5
0.6
0.7
0.8
0.9
1.0
1.1
1.2
1.3
1.4
1.5
Relative calibration accuracy
