# Supplementary material for: Synthetic auxotrophy reveals metabolic regulation of plasma cell generation, affinity maturation, and cytokine receptor signaling
Source: eLife. 2026 Oct 29;14:RP107627. doi: 10.7554/eLife.107627 (PMC13719169; doi:10.7554/eLife.107627)
Supplement: Supplementary file 17 [file elife-107627-fig4-figsupp2-data1.zip › source data - Figure 4, supplement 2/P169x2 - Detection in QCs and Samples.pdf]

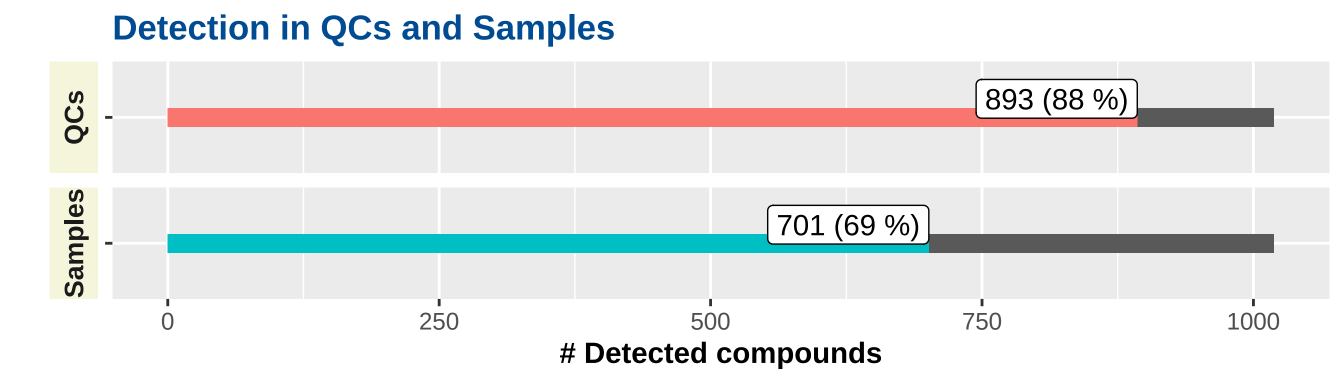
Detection in QCs and Samples
QCs
Samples
893 (88 %)
701 (69 %)
0
250
500
750
1000
Detected compounds
