# Supplementary material for: Synthetic auxotrophy reveals metabolic regulation of plasma cell generation, affinity maturation, and cytokine receptor signaling
Source: eLife. 2026 Oct 29;14:RP107627. doi: 10.7554/eLife.107627 (PMC13719169; doi:10.7554/eLife.107627)
Supplement: Supplementary file 17 [file elife-107627-fig4-figsupp2-data1.zip › source data - Figure 4, supplement 2/P169x2 - Detection in Samples by Class.pdf]

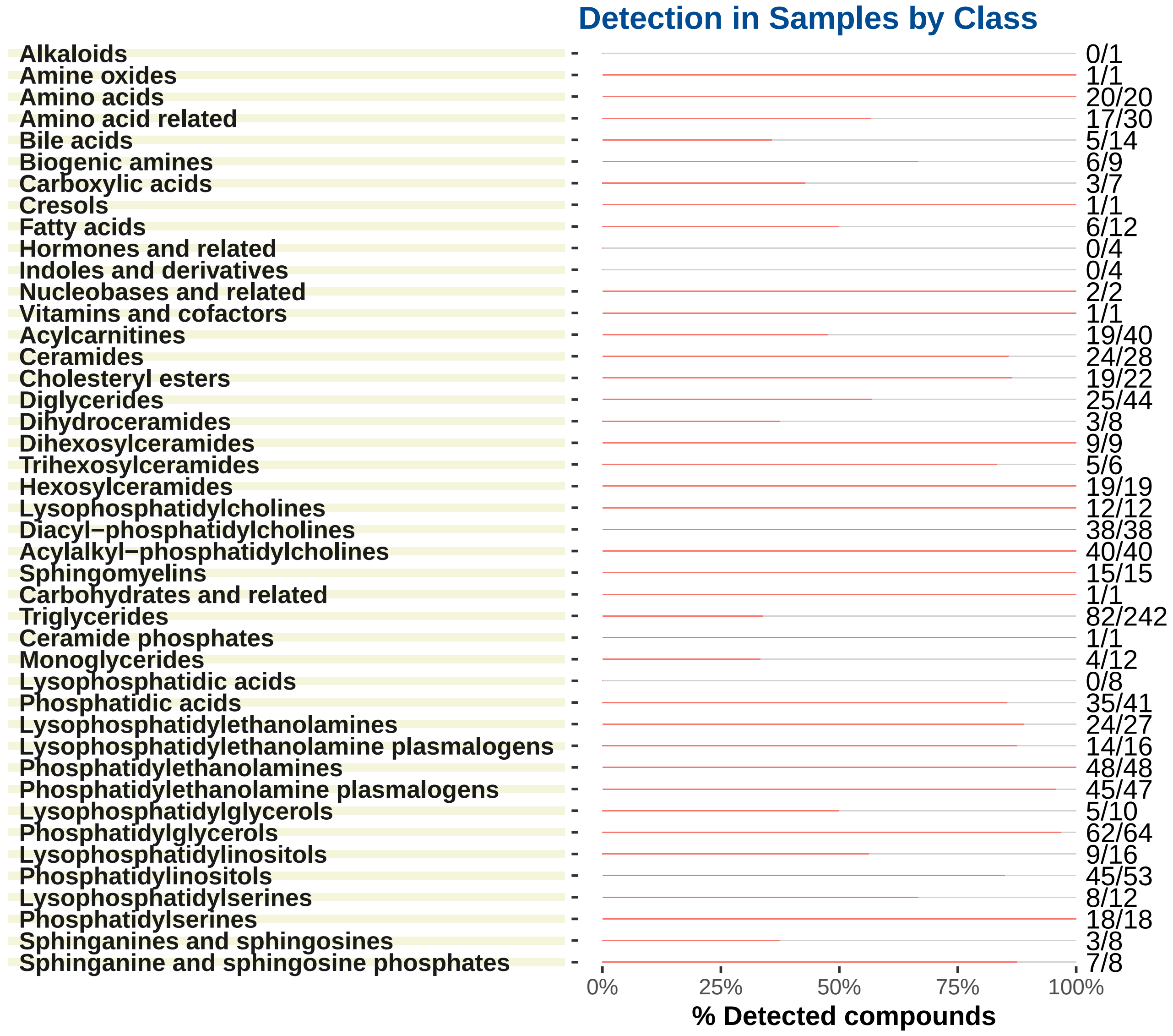
Detection in Samples by Class
Alkaloids 0/1
Amine oxides 1/1
Amino acids 20/20
Amino acid related 17/30
Bile acids 5/14
Biogenic amines 6/9
Carboxylic acids 3/7
Cresols 1/1
Fatty acids 6/12
Hormones and related 0/4
Indoles and derivatives 0/4
Nucleobases and related 2/2
Vitamins and cofactors 1/1
Acylcarnitines 19/40
Ceramides 24/28
Cholesteryl esters 19/22
Diglycerides 25/44
Dihydroceramides 3/8
Dihexosylceramides 9/9
Trihexosylceramides 5/6
Hexosylceramides 19/19
Lysophosphatidylcholines 12/12
Diacyl−phosphatidylcholines 38/38
Acylalkyl−phosphatidylcholines 40/40
Sphingomyelins 15/15
Carbohydrates and related 1/1
Triglycerides 82/242
Ceramide phosphates 1/1
Monoglycerides 4/12
Lysophosphatidic acids 0/8
Phosphatidic acids 35/41
Lysophosphatidylethanolamines 24/27
Lysophosphatidylethanolamine plasmalogens 14/16
Phosphatidylethanolamines 48/48
Phosphatidylethanolamine plasmalogens 45/47
Lysophosphatidylglycerols 5/10
Phosphatidylglycerols 62/64
Lysophosphatidylinositols 9/16
Phosphatidylinositols 45/53
Lysophosphatidylserines 8/12
Phosphatidylserines 18/18
Sphinganines and sphingosines 3/8
Sphinganine and sphingosine phosphates 7/8
0%
25%
50%
75%
100%
% Detected compounds
