# Supplementary material for: Synthetic auxotrophy reveals metabolic regulation of plasma cell generation, affinity maturation, and cytokine receptor signaling
Source: eLife. 2026 Oct 29;14:RP107627. doi: 10.7554/eLife.107627 (PMC13719169; doi:10.7554/eLife.107627)
Supplement: Supplementary file 17 [file elife-107627-fig4-figsupp2-data1.zip › source data - Figure 4, supplement 2/P169x2 - QC variation in detected compounds.pdf]

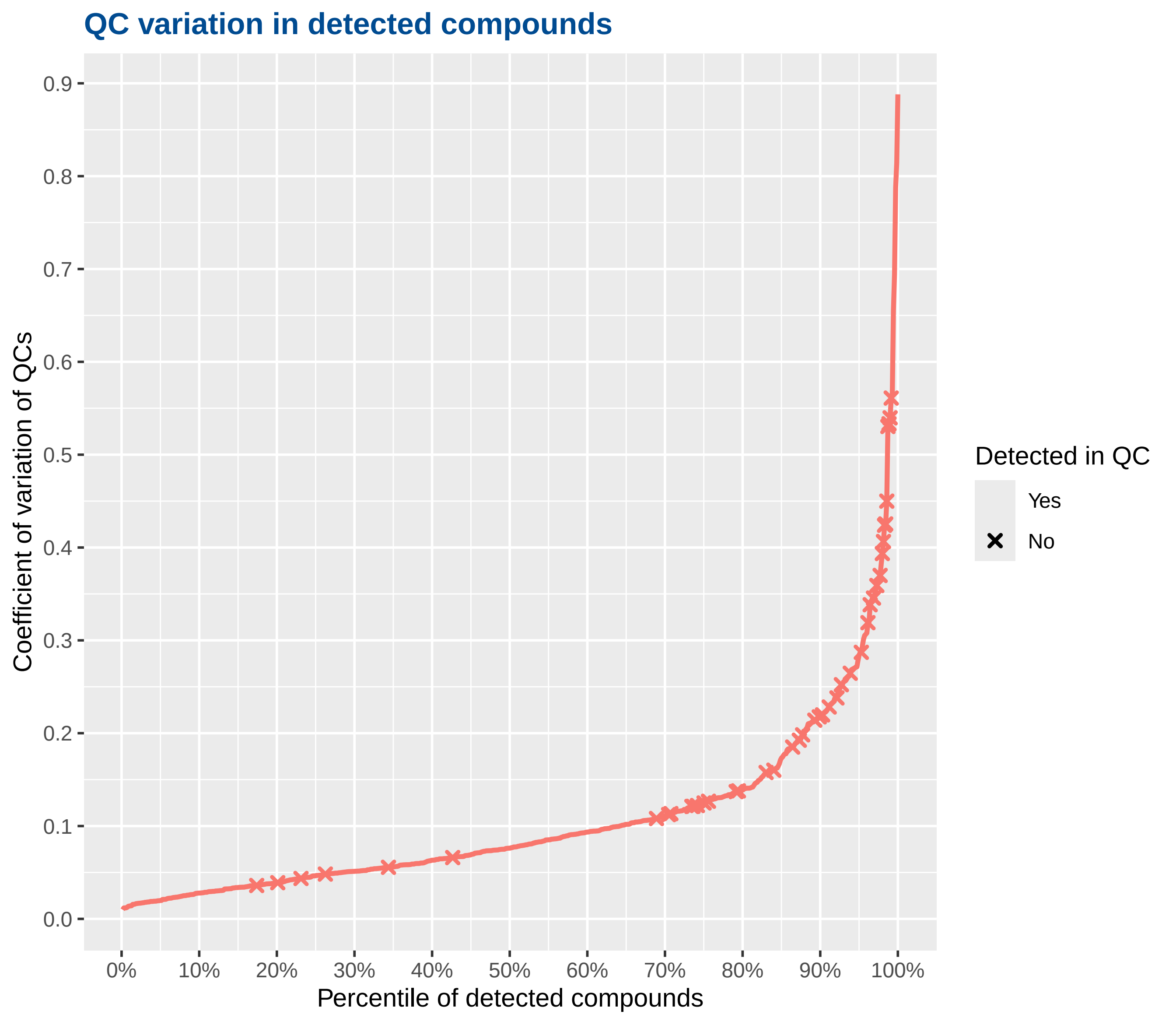

QC variation in detected compounds
Coefficient of variation of QCs
0.0
0.1
0.2
0.3
0.4
0.5
0.6
0.7
0.8
0.9
0%
10%
20%
30%
40%
50%
60%
70%
80%
90%
100%
Percentile of detected compounds
Detected in QC
Yes
No
