# Supplementary material for: Synthetic auxotrophy reveals metabolic regulation of plasma cell generation, affinity maturation, and cytokine receptor signaling
Source: eLife. 2026 Oct 29;14:RP107627. doi: 10.7554/eLife.107627 (PMC13719169; doi:10.7554/eLife.107627)
Supplement: Supplementary file 17 [file elife-107627-fig4-figsupp2-data1.zip › source data - Figure 4, supplement 2/P169x2 - QC variation in detected compounds by Class.pdf]

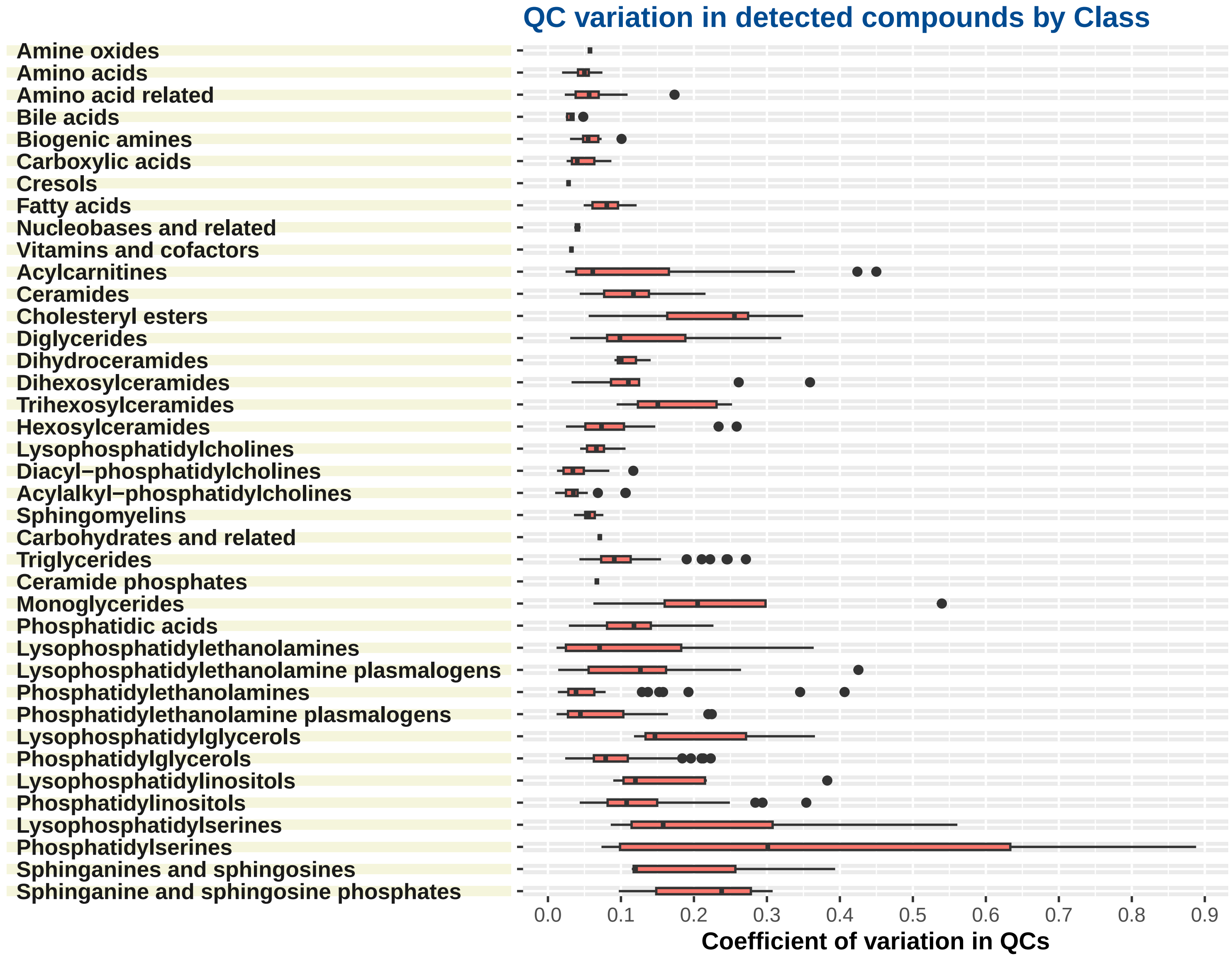
QC variation in detected compounds by Class
Amine oxides
Amino acids
Amino acid related
Bile acids
Biogenic amines
Carboxylic acids
Cresols
Fatty acids
Nucleobases and related
Vitamins and cofactors
Acylcarnitines
Ceramides
Cholesteryl esters
Diglycerides
Dihydroceramides
Dihexosylceramides
Trihexosylceramides
Hexosylceramides
Lysophosphatidylcholines
Diacyl−phosphatidylcholines
Acylalkyl−phosphatidylcholines
Sphingomyelins
Carbohydrates and related
Triglycerides
Ceramide phosphates
Monoglycerides
Phosphatidic acids
Lysophosphatidylethanolamines
Lysophosphatidylethanolamine plasmalogens
Phosphatidylethanolamines
Phosphatidylethanolamine plasmalogens
Lysophosphatidylglycerols
Phosphatidylglycerols
Lysophosphatidylinositols
Phosphatidylinositols
Lysophosphatidylserines
Phosphatidylserines
Sphinganines and sphingosines
Sphinganine and sphingosine phosphates
0.0
0.1
0.2
0.3
0.4
0.5
0.6
0.7
0.8
0.9
Coefficient of variation in QCs
