# Supplementary material for: Synthetic auxotrophy reveals metabolic regulation of plasma cell generation, affinity maturation, and cytokine receptor signaling
Source: eLife. 2026 Oct 29;14:RP107627. doi: 10.7554/eLife.107627 (PMC13719169; doi:10.7554/eLife.107627)
Supplement: Supplementary file 24 [file elife-107627-fig6-data1.zip › Figure 6b WB.pdf]

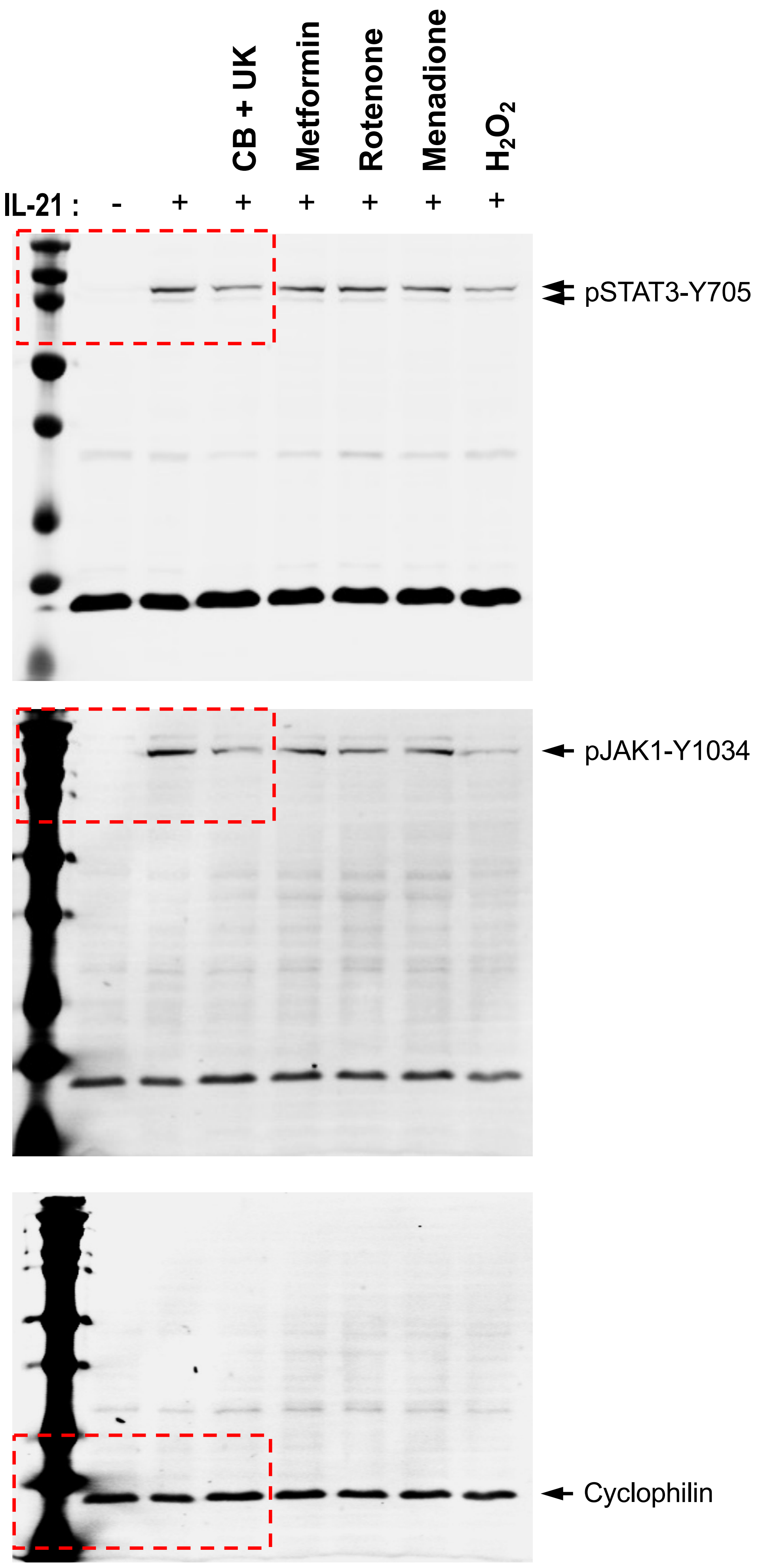
CB + UK
Metformin
Rotenone
Menadione
H_2O_2
IL-21 : - + + + + + +
pSTAT3-Y705
pJAK1-Y1034
Cyclophilin

Fig. 6b
