# Supplementary material for: Synthetic auxotrophy reveals metabolic regulation of plasma cell generation, affinity maturation, and cytokine receptor signaling
Source: eLife. 2026 Oct 29;14:RP107627. doi: 10.7554/eLife.107627 (PMC13719169; doi:10.7554/eLife.107627)
Supplement: Supplementary file 25 [file elife-107627-fig7-data1.zip › Figure 7e WB.pdf]

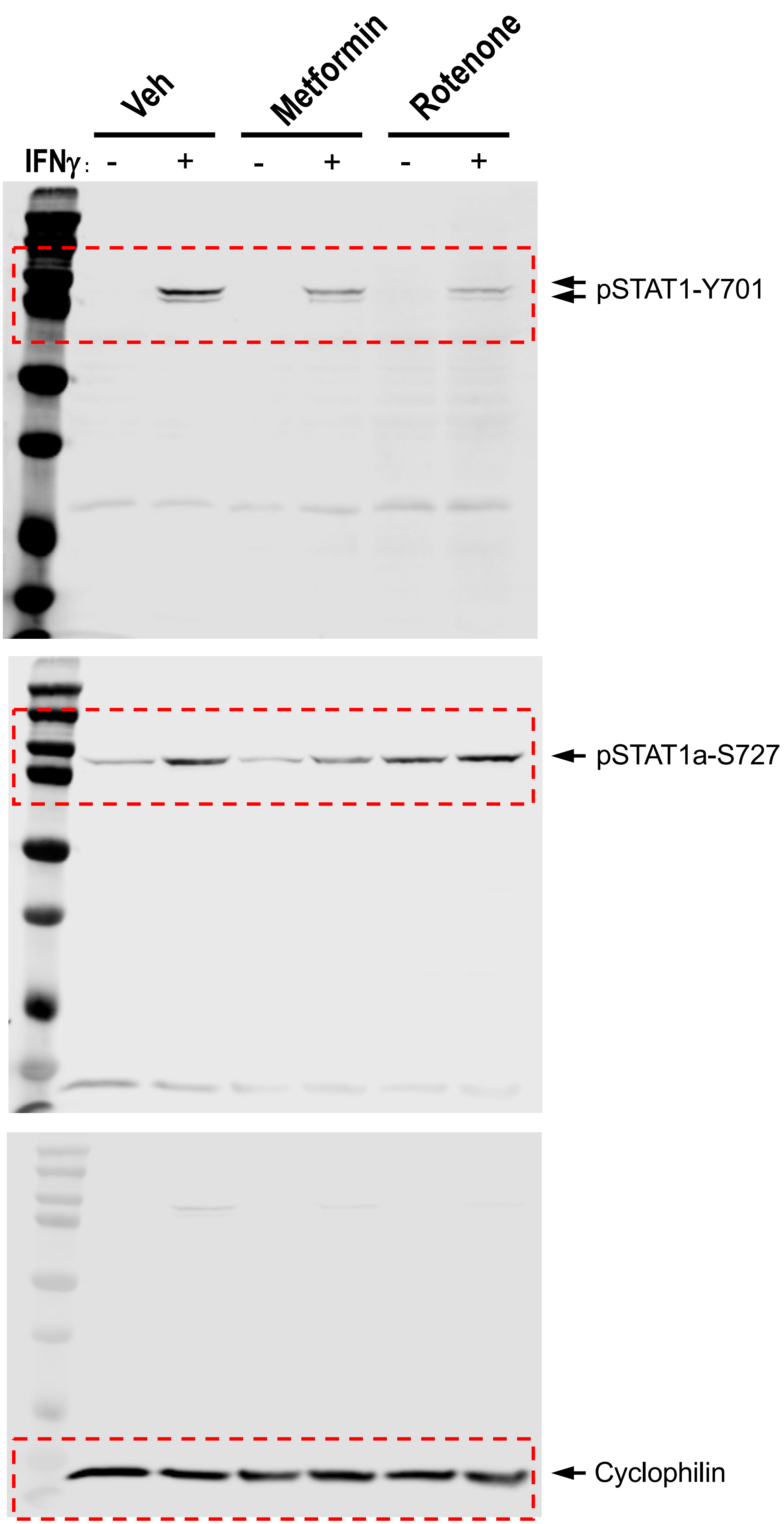
Veh
Metformin
Rotenone
IFNγ: - + - + - +
pSTAT1-Y701
pSTAT1a-S727
Cyclophilin

Fig. 7e
