# Supplementary material for: Synthetic auxotrophy reveals metabolic regulation of plasma cell generation, affinity maturation, and cytokine receptor signaling
Source: eLife. 2026 Oct 29;14:RP107627. doi: 10.7554/eLife.107627 (PMC13719169; doi:10.7554/eLife.107627)
Supplement: Supplementary file 26 [file elife-107627-fig7-figsupp1-data1.zip › Figure 7_Supplement 1d WB.pdf]

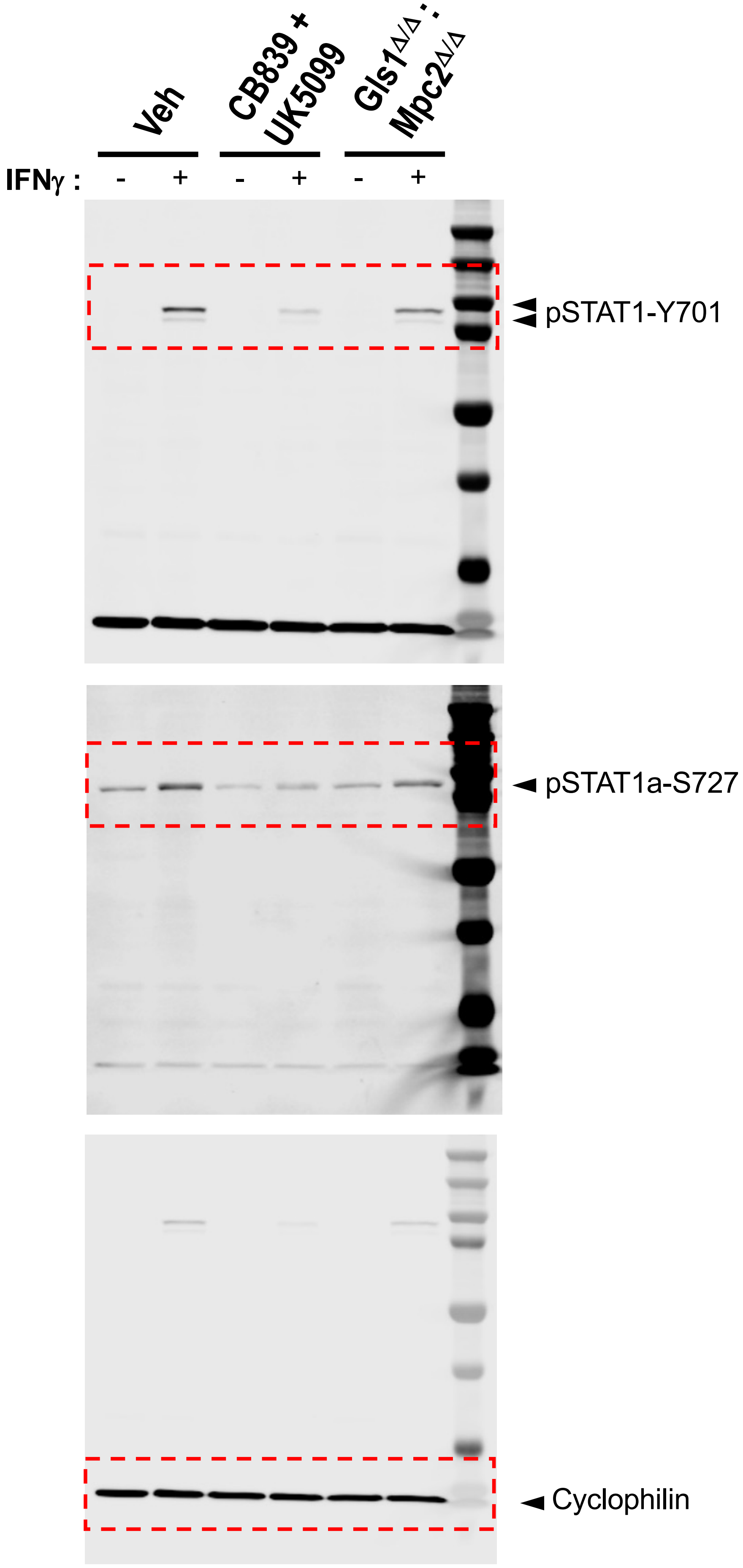

Fig. 7-Supplement 1d
