# Supplementary material for: Synthetic auxotrophy reveals metabolic regulation of plasma cell generation, affinity maturation, and cytokine receptor signaling
Source: eLife. 2026 Oct 29;14:RP107627. doi: 10.7554/eLife.107627 (PMC13719169; doi:10.7554/eLife.107627)
Supplement: Peer review history

# Editor report: eLife Assessment

**DOI:** 10.7554/eLife.107627.3.sa0

**Authors:** Tomohiro Kurosaki

**Affiliations:** The University of Osaka Japan

**Keywords:** Important, Solid

The authors addressed a significant biological question, namely the role of glutamine metabolism in humoral responses, and they obtained **solid** conclusions. The strength of this study is that the authors used state-of-the-art transgenic mouse models together with in vitro analysis, thereby providing **important** insights into the question posed. The manuscript has been further substantiated by adding more appropriate experimental controls and describing more in-depth functionality/physiological relevance.

# Referee report: Reviewer #2 (Public review)

**DOI:** 10.7554/eLife.107627.3.sa1

Summary:

In this manuscript, the authors investigate the functional requirements for glutamine and glutaminolysis in antibody responses. The authors first demonstrate that the concentrations of glutamine in lymph nodes are substantially lower than in plasma, and that at these levels, glutamine is limiting for plasma cell differentiation in vitro. The authors go on to use genetic mouse models in which B cells are deficient in glutaminase 1 (Gls), the glucose transporter Slc2a1, and/or mitochondrial pyruvate carrier 2 (Mpc2) to test the importance of these pathways in vivo. Interestingly, deficiency of Gls alone showed clear antibody defects when ovalbumin was used as the immunogen, but not the hapten NP. For the latter response, defects in antibody titers and affinity were observed only when both Gls and either Mpc2 or Slc2a1 were deleted. These latter findings form the basis of the synthetic auxotrophy conclusion. The authors go on to test these conclusions further using in vitro differentiations, Seahorse assays, pharmacological inhibitors, and targeted quantification of specific metabolites and amino acids. Finally, the authors document reduced STAT3 and STAT1 phosphorylation in response to IL-21 and interferon (both type 1 and 2), respectively, when both glutaminolysis and mitochondrial pyruvate metabolism are prevented.

Strengths:

(1) The main strength of the manuscript is the overall breadth of experiments performed. Orthogonal experiments are performed using genetic models, pharmacological inhibitors, in vitro assays, and in vivo experiments to support the claims. Multiple antigens are used as test immunogens--this is particularly important given the differing results.

(2) B cell metabolism is an area of interest but understudied relative to other cell types in the immune system.

(3) The importance of metabolic flexibility and caution when interpreting negative results is made clear from this study.

Weaknesses:

(1) All of the in vivo studies were done in the context of boosters at 3 weeks and recall responses 1 week later. Primary responses, including germinal centers, may still be ongoing at 3 weeks after the initial immunization and defects in GCs may contribute to the findings. Nonetheless, the authors do check antibody levels prior to the boost, and it is likely that most of the observed defects in Gls/Mpc2-deficiency are driven by faulty recall responses.

# Referee report: Reviewer #3 (Public review)

**DOI:** 10.7554/eLife.107627.3.sa2

Summary:

In their manuscript, the authors investigate how glutaminolysis (GLS) and mitochondrial pyruvate import (MPC2) jointly shape B cell fate and the humoral immune response. Using inducible knockout systems and metabolic inhibitors, they uncover a "synthetic auxotrophy": When GLS activity/glutaminolysis is lost together with either GLUT1-mediated glucose uptake or MPC2, B cells fail to upregulate mitochondrial respiration, IL 21/STAT3 and IFN/STAT1 signaling is impaired, and the plasma cell output and antigen-specific antibody titers drop significantly. This work thus demonstrates the promotion of plasma cell differentiation and cytokine signaling through parallel activation of two metabolic pathways. The dataset is technically comprehensive and conceptually novel, but some aspects leave the in vivo and translational significance uncertain.

Strengths:

(1) Conceptual novelty: the study goes beyond single-enzyme deletions to reveal conditional metabolic vulnerabilities and fate-deciding mechanisms in B cells.

(2) Mechanistic depth: the study uncovers a novel "metabolic bottleneck" that impairs mitochondrial respiration and elevates ROS and directly ties these changes to cytokine-receptor signaling. This is both mechanistically compelling and potentially clinically relevant.

(3) Breadth of models and methods: inducible genetics, pharmacology, metabolomics, seahorse assay, ELISpot/ELISA, RNA-seq, two immunization models.

(4) Potential clinical angle: the synergy of CB839 with UK5099 and/or hydroxychloroquine hints at a druggable pathway targeting autoantibody-driven diseases.

Comments on revised version.

Authors extensively modified the text with great care and provided new data e.g. Fig. 5. Collectively, this is convincing and hence, I have no further comments.

# Author comment: Author response

**DOI:** 10.7554/eLife.107627.3.sa3

**Authors:** Sung Hoon Cho, Shawna K Brookens, Ariel L Raybuck, Kaylor Meyer, Yeeun Paik, Jennie Hamilton, Jingxin Li, Karel Karel Kalecky, Chloe Park, Juyeon Lim, Sakeenah L Hicks, John Karijolich, Teodoro H Bottiglieri, Jeffrey Rathmell, Denis Mogilenko, Christopher D Scharer, Mark Boothby

**Affiliations:** Emory University Atlanta, GA United States; Vanderbilt University Medical Center Nashville United States; Vanderbilt University Medical Center Nashville United States; Vanderbilt University Medical Center Nashville United States; Vanderbilt University Medical Center Nashville United States; Vanderbilt University Medical Center Nashville United States; Vanderbilt University Medical Center Nashville United States; aylor Scott and White Research Institute Dallas United States; Vanderbilt University Medical Center Nashville United States; Vanderbilt University Medical Center Nashville, TN United States; Emory University Atlanta United States; Vanderbilt University Medical Center Nashville United States; Baylor Scott and White Research Institute Dallas United States; Vanderbilt University Medical Center Nashville United States; Vanderbilt University Medical Center Nashville United States; Emory University Atlanta United States; Vanderbilt University Medical Center Nashville United States

The following is the authors’ response to the current reviews.

We thank the referees for noting the substantive revisions and for the praise of the work. While we each have somewhat different weightings of likelihood, we feel the appraisals are fair and reasonable.

The following is the authors’ response to the original reviews.

**eLife Assessment**

The authors addressed an important biological question, namely the role of glutamine metabolism in humoral responses, and they obtained solid conclusions. The strength of this study is that the authors used state-of-the-art transgenic mouse models together with in vitro analysis, thereby providing significant insights into the question posed. The following would strengthen the manuscript: (i) adding more in-depth functionality/physiological relevance in the discussion part, and (ii) regarding the experiments, the inclusion of more appropriate controls and a clearer and more accurate description of the methods.

We are grateful for the decision of the Editors to select this submission for in-depth peer review and to the Reviewing Editor and referees for the thoughtful and constructive comments.

We mostly agree with the specific comments and evaluation of strengths of what the work adds as well as with indications of limitations and caveats that apply to the breadth of conclusions. We have edited the text to be more clear and provide more details about certain aspects of the Methods and Legends. In addition, although we try to avoid Discussion sections that are unduly long or have flights of fancy, we will add to the Discussion as well as edit it for directness about potential relevance, basic explorations of mechanisms, and functionality.

The revised manuscript also contains new data, some of it dealing with comments of the referees, other additions representing work done while the manuscript was under review. While we would be inclined to do more, the sad practical problem is one of limits placed by both the absence of any grant funds and the institution's terminations (RIFs) of the two experimenters in the lab.

While we believe the original data interpretable as presented originally, up to a point it nonetheless is good to enhance scope or have even better data and add refinements about some of the technical issues. Ultimately, the question becomes "when is enough enough?"

In the detailed point-by-point response below, we outline changes prompted by the reviewers. We also comment on a few points more expansively that would be suitable for the paper itself, and offer some skepticism or disagreement, longer and more detailed explanations.

**Public Reviews:**

**Reviewer #1 (Public review):**

Summary:

In this manuscript, Cho et al. present a comprehensive and multidimensional analysis of glutamine metabolism in the regulation of B cell differentiation and function during immune responses. They further demonstrate how glutamine metabolism interacts with glucose uptake and utilization to modulate key intracellular processes. The manuscript is clearly written, and the experimental approaches are informative and well-executed. The authors provide a detailed mechanistic understanding through the use of both in vivo and in vitro models. The conclusions are well supported by the data, and the findings are novel and impactful. I have only a few, mostly minor, concerns related to data presentation and the rationale for certain experimental choices.

Detailed Comments:

(1) In Figure 1b, it is unclear whether total B cells or follicular B cells were used in the assay. Additionally, the in vitro class-switch recombination and plasma cell differentiation experiments were conducted without BCR stimulation, which makes the system appear overly artificial and limits physiological relevance. Although the effects of glutamine concentration on the measured parameters are evident, the results cannot be confidently interpreted as true plasma cell generation or IgG1 class switching under these conditions. The authors should moderate these claims or provide stronger justification for the chosen differentiation strategy. Incorporating a parallel assay with anti-BCR stimulation would improve the rigor and interpretability of these findings.

We edited the manuscript to be clear that total splenic B cells were used in this set-up figure and the rest of the paper. In addition, we performed new experiments to improve this "set-up figure (Fig. 1)" and moved the older data using alternative experimental conditions to a supplemental figure, Figure 1 - supplement 1. We also used new conditions that included styles of stimulating proliferation and differentiation - to foster an increased sense of generality. The findings in no way change the supported conclusions of the work. Specifically, we used mitogenic stimulation with anti-IgM ^+^ anti-CD40, all with BAFF, IL-4, and IL-5 in addition to the anti-CD40 stimulation of the original manuscript, bearing in mind excellent work from Aiba et al, Immunity 2006; 24: 259-268, and similar papers. In addition, we added a panel with representative flow cytometric profiles. These new data are presented in Figure 4 - supplement 1 (panels ae).

To be transparent and add to a more open public discussion (using the virtues of this forum), the senior author and colleagues would caution about whether any in vitro conditions exist that warrant complete confidence. That is the reason for proceeding to immunization experiments *in vivo*. That is not said to cast doubt on our own in vitro data - there are some experiments (such as those of Fig. 1a-c and associated Fig 1 - supplement 1) that only can be done in vitro or are better done that way (e.g., because of rapid uptake of early apoptotic B cells *in vivo*).

For instance: Well-respected papers use the CD40LB and NB21.2D9 systems to activate B cells and generate plasma cells. Those appear to be BCR-independent and yet continue in common use. [We found that these cellular systems (CD40LB; NB21.2D9) cannot be used in experiments with a.a. deprivation or the inhibitors due to effects on the engineered stroma-like cells.] In considering BCR engagement, Reth has published salient points about signaling and concentrations of the Ab, the upshot being that this means of activating mitogenesis and plasma cell differentiation when the B cells are costimulated via CD40 or TLR (4 or 7/8) is also artificial. Moreover, although Aiba et al, Immunity 2006; 24: 259-268 is a laudable exception, one rarely finds papers using BAFF despite the strong evidence it is an essential part of the equation of B cell regulation *in vivo* and a cytokine that modulates BCR signaling - in the cultures.

(2) In Figure 1c, the DMK alone condition is not presented. This hinders readers' ability to properly asses the glutaminolysis dependency of the cells for the measured readouts. Also, CD138^+^ in developing PCs goes hand in hand with decreased B220 expression. A representative FACS plot showing the gating strategy for the in vitro PCs should be added as a supplementary figure. Similarly, division number (going all the way to #7) may be tricky to gate and interpret. A representative FACS plot showing the separation of B cells according to their division numbers and a subsequent gating of CD138 or IgG1 in these gates would be ideal for demonstrating the authors' ability to distinguish these populations effectively.

In the revised manuscript, we have added new experimental data (Figure 1).

We agree that exact placement of divisions and deconvolution by FlowJow is more fraught than might be thought from presentations in many or most papers. We include the data shown to the right as representative FACS plot(s) with old and new data that illustrate the gating on CTV fluorescence. With the representative examples pasted in here and presented in Fig 1 - supplement 1f, g of the revised manuscript, we will aver that using divisions 0-6, and ≥7 was and is entirely reasonable.

Ditto for DMK with normal glutamine. However, in the spirit of eLife transparency lacking in many other journals, this comparison is more fraught than the referee comment would make things seem. The concentration tolerated by cells is highly dependent on the medium and glutamine concentration, and perhaps on rates of glutaminolysis (due to its generation of ammonia). In practice, DMK becomes more toxic to B cells unless glutamine is low or glutaminolysis is restricted. Thus, the concentration of DMK that is tolerated and used in Fig. 1b, c can become toxic to the B cells when using the higher levels of glutamine in typical culture media (2 mM or more) - at which point the "normal conditions ^+^ DMK" "control" involves the surviving cells in conditions with far greater cell death and less population expansion than the "low glutamine ^+^ DMK". condition.

(3) A brief explanation should be provided for the exclusive use of IgG1 as the readout in classswitching assays, given that naïve B cells are capable of switching to multiple isotypes. Clarifying why IgG1 was preferentially selected would aid in the interpretation of the results.

On lines ~112-3 and ~182-5, we edited the text in light of the referee's suggestion that we focus the presentation of serologic data on IgG1 in the immunization experiments. We also rearranged figures and panels to be more explicit and harmonize. That said, and [Brief explanation - IgG1 provides the strongest signal and hence better signal/noise both in vitro and with the alum-based immunizations that are avatars for the adjuvant used in the majority of protein-based vaccines for humans. Perhaps for this reason, the majority of papers on molecular mechanisms seem only to analyze IgG1. Nonetheless, since molecular regulation can differ according to isotype, and the more pro-inflammatory mouse IgG2c is more pertinent to some forms of anti-pathogen immunity and some auto-immune disease models, we believe it valuable to retain these data in supplements to the related Figures.]

(4) The immunization experiments presented in Figures 1 and 2 are well designed, and the data are comprehensively presented. However, to prevent potential misinterpretation, it should be clarified that the observed differences between NP and OVA immunizations cannot be attributed solely to the chemical nature of the antigens - hapten versus protein. A more significant distinction lies in the route of administration (intraperitoneal vs. intranasal) and the resulting anatomical compartment of the immune response (systemic vs. lung-restricted). This context should be explicitly stated to avoid overinterpretation of the comparative findings.

We appreciate the positive assessment, and agree with the referee that it is possible the conditions of immune challenge or re-exposure may contribute to the observed differences. We edited the text of the revised manuscript accordingly [lines ~152-153; ~159-160]. Certainly, the difference in how the anti-ova response is elicited compared to the anti-NP response in the same mice or with a bit different an immunization regimen might be another factor - or the major factor - explaining why glutaminolysis was important after ovalbumin inhalations (used because emergence of anti-ova Ab / ASCs is suppressed by the NP hapten after NP-ova immunization) but not needed for the anti-NP response unless *Slc2a1* or *Mpc2* also was inactivated. Thank you prompting addition of this important caveat!

Nevertheless, it seems fair to note that in Figures 1 and 2, the ASCs and Ab are being analyzed for NP and ova in the same mice, albeit with the NP-specific components not being driven by the inhalations of ovalbumin. With that in mind, when one compares the IgG1 anti-NP ASC and Ab to those for IgG1 anti-ovalbumin (ASC in bone marrow; Ab), the ovalbumin-specific response was reduced whereas the anti-NP response was not. [lines ~171-172]

(5) NP immunization is known to be an inducer of an IgG1-dominant Th2-type immune response in mice. IgG2c is not a major player unless a nanoparticle delivery system is used. However, the authors arbitrarily included IgG2c in their assays in Figures 2 and 3. This may be confusing for the readers. The authors should either justify the IgG2c-mediated analyses or remove them from the main figures. (It can be added as supplemental information with proper justification).

We rearranged the Figure panels to move IgM and IgG2c data to Supplemental Figures (Figure 3 - supplements 1, 2, 4, 5 in the eLife system).

For purposes of public discourse, we note first that in contrast to the premise about weak IgG2c responses, the data [previously, Figure 3(c, g); now in the supplements] show substantial levels of NP-specific IgG2c. The referee is quite right that the class switching and in vitro ASC generation were done with IL-4 / IgG1-promoting conditions.

To assist readers, the revised manuscript takes note of the important role of IgG2c (mouse - IgG1 in humans) in controlling or clearing various pathogens as well as in autoimmunity [lines ~182-5]. Moreover, we continue to think that these measurements add substantial value both from the standpoint of providing a better sense of generality to the loss-of-function effects, and in considering potential ways of translating the findings to B cell-dependent autoimmune conditions such as systemic lupus erythematosus.

As a scientific aside, we speculate that a greater or lesser IgG2c anti-NP response may arise due to different preparations of NP-carrier obtained from the vendor (Biosearch) having different amounts of TLR (e.g., TLR4) ligand. In any case, the points of presenting the IgG2c (and IgM) data were to push against the limiting boundaries of convention (which risks perpetuating a narrow view of potential outcomes) and make the breadth of results more apparent to readers.

(6) Similarly, in affinity maturation analyses, including IgM is somewhat uncommon. I do not see any point in showing high affinity (NP2/NP20) IgMs (Figure 3d), since that data probably does not mean much.

As noted in the reply immediately preceding this one, we appreciate this suggestion from the reviewer and moved the IgM and IgG2c to supplemental status.

Nonetheless, in collegial discourse we disagree a bit with the referee in light of our data as well as of work that (to our minds) leads one to question why inclusion of affinity maturation of IgM is so uncommon - as the referee accurately notes. Of course a defect in the capacity to class-switch is highly deleterious in patients but that is not the same as concluding that recall IgM or its affinity is of little consequence.

In some of the pioneering work back in the 1980's, Bothwell showed that NP- carrier immunization generated hybridomas producing IgM Ab with extensive SHM (~11% of the 18 lineages; ~ 1/3 of the IgM hybridomas) [PMID: 8487778], IgM B cells appear to move into GC, and there is at least a reasonable published basis for the view that there are GC-derived IgM (unswitched) memory B cells (MBC) that would be more likely, upon recall activation, to differentiate into ASCs. As an example, albeit with the Jenkins lab anti-rPE response, Taylor, Pape, and Jenkins generated quantitative estimates of the numbers of Ag-specific IgM^+^ vs switched MBC that were GC-derived (or not). [PMID: 22370719]. While they emphasized that ~90% of IgM^+^ MBC appeared to be GC-independent, their data also indicated that ~1/2 of all GC-derived MBC were IgM^+^ rather than switched (their Fig. 8, B vs C; also 8E, which includes alum-PE). And while we immensely respect the referee, we are perhaps less confident that IgM or high-affinity Ag-specific IgM doesn't mean that much, if only because of evidence that localized Ab compete for Ag and may thus influence selective processes [PMCID: PMC2747358; PMID: 15953185; PMID: 23420879; PMID: 27270306].

(7) Following on my comment for the PC generation in Figure 1 (see above), in Figure 4, a strategy that relies solely on CD40L stimulation is performed. This is highly artificial for the PC generation and needs to be justified, or more physiologically relevant PC generation strategies involving anti-BCR, CD40L, and various cytokines should be shown.

In line with our response to point (1), we tested BCR-stimulated B cells (anti-CD40 plus anti-IgM with BAFF, IL-4, and IL-5, parallel to the analyses with anti-CD40 but no BCR engagement). These results align with and reinforce the utility of the data with anti-CD40 as the sole mitogen.

(8) The effects of CB839 and UK5099 on cell viability are not shown. Including viability data under these treatment conditions would be a valuable addition to the supplementary materials, as it would help readers more accurately interpret the functional outcomes observed in the study.

We added presentation of data that provide cues as to relative viability / cxmsurvival under the experimental conditions used.

[FSC X SSC as well as 7AAD or Ghost dye panels; we also generated new data that in further experiments scoring annexin V staining (see Fig 4 - supplement 1d, e, and Fig 5 - supplement 1e, f)].

(9) It is not clear how the RNA seq analysis in Figure 4h was generated. The experimental strategy and the setup need to be better explained.

Including text added at lines ~291-293 and ~582-585, the revised manuscript provides more information in the Results, Methods and Legend for Fig 4j-l. We agree entirely with the concern and apologize that in this and a few other instances we inadvertently sacrificed sufficiency of detail on the altar of attempting brevity.

As a synopsis: In three temporally and biologically independent experiments, cultures were harvested 3.5 days after splenic B cells were purified and cultured as in the experiments of Fig. 4a-e. Total cellular RNA was prepared from the twelve samples (three replicates for each of four conditions - DMSO vehicle control, CB839, UK5099, and CB839 ^+^ UK5099), then analyzed by RNA-seq. RNA-seq data were initially processed using the pipeline described in the Methods. For panels g & h of Fig 4, DESeq2 was used to quantify and compare read counts in the three CB839 ^+^ UK5099 samples relative to the three independent vehicle controls and identify all genes for which variances yielded P<0.05. In Fig 4g, all such genes for which the difference was 'statistically significant' (i.e., P<0.05) were entered into the indicated Immgen tool and thereby mapped to the B lineage subsets shown in the figure panels (i.e., g, h). In (*g*), these are displayed using one format, whereas (*h*) uses the 'heatmap' tool in MyGeneSet.

**Reviewer #2 (Public review):**

Summary:

In this manuscript, the authors investigate the functional requirements for glutamine and glutaminolysis in antibody responses. The authors first demonstrate that the concentrations of glutamine in lymph nodes are substantially lower than in plasma, and that at these levels, glutamine is limiting for plasma cell differentiation in vitro. The authors go on to use genetic mouse models in which B cells are deficient in glutaminase 1 (Gls), the glucose transporter Slc2a1, and/or mitochondrial pyruvate carrier 2 (Mpc2) to test the importance of these pathways in vivo.

Interestingly, deficiency of Gls alone showed clear antibody defects when ovalbumin was used as the immunogen, but not the hapten NP. For the latter response, defects in antibody titers and affinity were observed only when both Gls and either Mpc2 or Slc2a1 were deleted. These latter findings form the basis of the synthetic auxotrophy conclusion. The authors go on to test these conclusions further using in vitro differentiations, Seahorse assays, pharmacological inhibitors, and targeted quantification of specific metabolites and amino acids. Finally, the authors document reduced STAT3 and STAT1 phosphorylation in response to IL-21 and interferon (both type 1 and 2), respectively, when both glutaminolysis and mitochondrial pyruvate metabolism are prevented.

Strengths:

(1) The main strength of the manuscript is the overall breadth of experiments performed. Orthogonal experiments are performed using genetic models, pharmacological inhibitors, in vitro assays, and in vivo experiments to support the claims. Multiple antigens are used as test immunogens--this is particularly important given the differing results.

(2) B cell metabolism is an area of interest but understudied relative to other cell types in the immune system.

(3) The importance of metabolic flexibility and caution when interpreting negative results is made clear from this study.

Weaknesses:

(1) All of the in vivo studies were done in the context of boosters at 3 weeks and recall responses 1 week later. This makes specific results difficult to interpret. Primary responses, including germinal centers, are still ongoing at 3 weeks after the initial immunization. Thus, untangling what proportion of the defects are due to problems in the primary vs. memory response is difficult.

We performed new experiments and added the data on differences prior to a boost [see below; new Fig 3d, e; etc].

(2) Along these lines, the defects shown in Figure 3h-i may not be due to the authors' interpretation that Gls and Mpc2 are required for efficient plasma cell differentiation from memory B cells. This interpretation would only be correct if the absence of Gls/Mpc2 leads to preferential recruitment of low-affinity memory B cells into secondary plasma cells. The more likely interpretation is that ongoing primary germinal centers are negatively impacted by Gls and Mpc2 deficiency, and this, in turn, leads to reduced affinities of serum antibodies.

We have edited the wording of the conclusion to add a possibility we consider unlikely and downplay a conclusion that MBCs bearing switched BCRs are affected once reactivated. [see lines ~221-230] We also have added citations pertaining to the topic, including work from the Victora lab which seems to put the point succinctly: "Recall GCs in mice consist almost entirely of naïve B cells, whereas recall antibodies derive overwhelmingly from memory B cells." [emphasis added] [PMID: 38838672; new ref #83]. While unclear as to the reasoning - as one looks at the data - and skeptical as to the accuracy of the referee's point (2), it suggests that the matter is open to reasonable doubt. In line with the point and the edits, we also have added citation of a bioRxiv preprint from the Victora lab, which touches on the concept of what one could call boost-induced reinvigoration of a pre-existing GC [new ref #82].

Beyond the textual changes, we performed a new series of experiments to investigate partially, and present the results in Fig 3d, e as well as Fig 3 - supplement 1d, e. Unfortunately, time before lab closure was an enemy both for the period between primary and recall immunizations in performance and multiple replication of work to extend that presented in Figure 3, panels g & h, and the related Supplemental Data (Fig 3 - supplements 4d, 5a-g). Unfortunately, it was not possible to do a longer-term memory experiment with recall immunization out at 8 weeks.

The intriguing concerns and questions of points 1 & 2 provide a springboard for consideration of generalizations and simplifications. Germinal center durability is not at all monolithic, and instead is quite variable**. It is true that in the literature (especially with the substantially different approach of transferring BCR-transgenic / knock-in versions of an NP-biased BCR) there may be meaningful pools of IgG1 and IgG2c GC B cells. The premise (cognitive bias, perhaps?) in our interpretation is that in our previous work we measured few if any GC B cells - NP-APC-binding or otherwise - above the background (non-immunized controls) three weeks after immunization with NP-ovalbumin in alum. While recognizing that the immunogen can matter, we note for the readers and referee that Fig. 1 of the Taylor, Pape, & Jenkins paper considered above [PMID: 22370719] reported 10-fold more Ag-specific MBCs than GC B cells at day 29 post-immunization the point at which the boost/recall challenge was performed in our Figure 3g, h. [That work did not use NP-carrier in alum to immunize, or measure the anti-NP response.]

Viewing Fig. 3i from that perspective, the surmise of the comment is that a major contribution to the differences in both all-affinity and high-affinity anti-NP IgG1 (whose production requires differentiation into plasma cells) derived from the immunization at 4 wk stimulating persistent GC B cells as opposed to memory B cells.

The issue and question also relate to rates of output of plasma cells or rises in the serum concentrations of class-switched Ab. To this point, our prior experiences agree with the long-published data of the Kurosaki lab in Figure 3c of the Aiba et al paper noted above (Immunity, 2006) (and other such time courses). Readers can note that the IgG1 anti-NP response (alum adjuvant, as in our work) hits its plateau at 2 wk, and did not increase further from 2 to 3 wk. The most likely interpretation is that GC are on the decline and Ab production has reached its plateau by the time of the 2nd immunization in Fig. 3h.

Assuming we understand the comment and line of reasoning correctly, we also lean towards disagreeing with the statement " This interpretation would only be correct if the absence of Gls/Mpc2 leads to preferential recruitment of low-affinity memory B cells into secondary plasma cells. Our evidence shows that both low-affinity as well as high-affinity anti-NP Ab (IgG1) were reduced due to combined gene-inactivation after the peak primary response (Fig. 3h; also, see the new data in Fig 3 and Fig 3 - supplement 1). Recent papers show that affinity maturation is attributable to greater proliferation of plasmablasts with high-affinity BCR. Accordingly, the findings with loss of GLS and MPC function are quite consistent with the interpretation that much of the response after the second immunization draws on MBC differentiation into plasmablasts and then plasma cells, where the proliferative advantage of high-affinity cells is blunted by the impaired metabolism. Notwithstanding these issues, the revised manuscript includes the alternative, if less likely, interpretation proposed by the review [lines ~221-230].

**In some contexts, of course, especially certain viral infections or vaccination with lipid nanoparticles carrying modified mRNA, germinal centres are far more persistent; also, in humans even the seasonal flu vaccine

(3) The gating strategies for germinal centers and memory B cells in Supplemental Figure 2 are problematic, especially given that these data are used to claim only modest and/or statistically insignificant differences in these populations when Gls and Mpc2 are ablated. Neither strategy shows distinct flow cytometric populations, and it does not seem that the quantification focuses on antigen-specific cells.

The revised manuscript improves these aspects of the presentation, using old and new data. See Fig 3 - supplement 3a, c; Fig 3 - supplement 4a. We note for readers that many other papers in the best journals show plots in which the separation of, say, GC-Tfh from overall Tfh is based on cut-off within what essentially is a continuous spectrum of emission as adjusted or compensated by the cytometer (spectral or conventional).

The revised manuscript presents results from new experiments that deal with the subset of GC B cells whose BCRs bind NP-APC with enough affinity to retain a positive signal after washing. These new data are presented in Fig 3 - supplement 3c & 3e. In practice, the new findings suggest that the metabolic requirement applied more to the NP-binding B cells than the overall GC B cell population.

(4) Along these lines, the conclusions in Figure 6a-d may need to be tempered if the analysis was done on polyclonal, rather than antigen-specific cells. Alum induces a heavily type 2-biased response and is not known to induce much of an interferon signature. The authors' observations might be explained by the inclusion of other ongoing GCs unrelated to the immunization.

We apologize for ambiguity or insufficient clarity and, as noted above, have edited the text to be more clear that the in vitro experiments do not represent GC B cells and that the RNA-seq data were from experiments that did not involve alum and were not an Ag (SRBC)-specific subset.

New text in the Results, an expanded Legend, and tweaking the Methods make it more readily clear that the RNA-seq data (and hence the GSEA) involved immunizations with SRBC (not the alum / NP system. That said, we note that the hapten-carrier experiments in which the immunogen was adjuvantized with alum actually generated a robust IgG2c type 1-driven) response along with the type 2-enhanced IgG1 response, in line with what has been reported by others with alum-adjuvanted vaccination.

**Reviewer #3 (Public review):**

Summary:

In their manuscript, the authors investigate how glutaminolysis (GLS) and mitochondrial pyruvate import (MPC2) jointly shape B cell fate and the humoral immune response. Using inducible knockout systems and metabolic inhibitors, they uncover a "synthetic auxotrophy": When GLS activity/glutaminolysis is lost together with either GLUT1-mediated glucose uptake or MPC2, B cells fail to upregulate mitochondrial respiration, IL 21/STAT3 and IFN/STAT1 signaling is impaired, and the plasma cell output and antigen-specific antibody titers drop significantly. This work thus demonstrates the promotion of plasma cell differentiation and cytokine signaling through parallel activation of two metabolic pathways. The dataset is technically comprehensive and conceptually novel, but some aspects leave the in vivo and translational significance uncertain.

Strengths:

(1) Conceptual novelty: the study goes beyond single-enzyme deletions to reveal conditional metabolic vulnerabilities and fate-deciding mechanisms in B cells.

(2) Mechanistic depth: the study uncovers a novel "metabolic bottleneck" that impairs mitochondrial respiration and elevates ROS, and directly ties these changes to cytokinereceptor signaling. This is both mechanistically compelling and potentially clinically relevant.

(3) Breadth of models and methods: inducible genetics, pharmacology, metabolomics, seahorse assay, ELISpot/ELISA, RNA-seq, two immunization models.

(4) Potential clinical angle: the synergy of CB839 with UK5099 and/or hydroxychloroquine hints at a druggable pathway targeting autoantibody-driven diseases.

We agree and thank the referee for the positive comments and this succinct summary of what we view as contributions of the paper.

Weaknesses:

(1) Physiological relevance of "synthetic auxotrophy"

The manuscript demonstrates that GLS loss is only crippling when glucose influx or mitochondrial pyruvate import is concurrently reduced, which the authors name "synthetic auxotrophy". I think it would help readers to clarify the terminology more and add a concise definition of "synthetic auxotrophy" versus "synthetic lethality" early in the manuscript and justify its relevance for B cells.

We edited the Abstract, Introduction, and Discussion to try to do better on this score. Conscious of how expansive the prose and data are even in the original submission, we appear to have taken some shortcuts that we will try to rectify or at least mitigate. Thank you for highlighting this need to improve on key concepts !!

Specifically, the revised text expands a bit on the notion that synthetic auxotrophy represents effects on differentiation that go beyond additional mechanisms of reducing division efficiency and a modest impact on selective death. [see the 10th - 11th lines in Abstract and lines ~84-85, Introduction] Even though decreased population expansion is observed and new evidence supports a model in which the altered metabolism contributes to enhanced death *in vivo*, at equal division numbers the frequency of CD138^+^ progeny is lower once glutaminolysis and mitochondrial pyruvate are reduced by either genetic or pharmacological means.

This comment of the review raises interesting semantic questions about what represents "physiological relevance". The fundamental point is to explore a basic science question - what, if any, are limits to metabolic flexibility? In principle, shouldn't B cells be able to use fatty acid metabolism to generate enough ATP and provide the backbones for biosynthesis during growth? Put a different way, the point is that a basic curiosity to understand why decreasing glucose influx did not have an even more profound effect than what was observed, combined with curiosity as to why glutaminolysis was dispensable in relatively standard vaccine-like models of immunize/boost, provided a springboard to identification of new vulnerabilities. The manuscript shows one physiological limitation (and hence vulnerability). Be that as it may, the revised text of the Discussion section more clearly addresses this issue (lines ~531-549 at the end of the Discussion).

While the overall findings, especially the subset specificity and the clinical implications, are generally interesting, the "synthetic auxotrophy" condition feels a little engineered.

CAR-T cells are 'a little engineered' (or more than a little) and yet they do seem to have had an impact on understanding the centrality of B cells in various autoimmune conditions as well as in the direction of cancer therapy research. So it is a matter of balancing this perspective of the referee against the strengths they highlight in points 1, 2, and 4. In editing the revision, we try to expand and be more explicit about this in the Discussion of the revised manuscript.

In brief, even were the money not all gone, we would not believe that expanding the heft of this already rather large manuscript and set of data would be appropriate. As matters stand, a basic new insight about metabolic flexibility and its limits leads to evidence of a way to reduce generation of Ab and a novel impairment of STAT transcription factor induction by several cytokine receptors. The vulnerability that could be tested in later work on B cell-dependent autoimmunity includes the capacity to test a compound that already has been to or through FDA phase II in patients together with an FDA-approved standard-of-care agent.

Therefore, the findings strongly raise the question of the likelihood of such a "double hit" in vivo and whether there are conditions, disease states, or drug regimens that would realistically generate such a "bottleneck".

Hence, the authors should document or at least discuss whether GC or inflamed niches naturally show simultaneous downregulation/lack of glutamine and/or pyruvate. The authors should also aim to provide evidence that infections (e.g., influenza), hypoxia, treatments (e.g., rapamycin), or inflammatory diseases like lupus co-limit these pathways.

Again, we appreciate some 'licensing' to be more expansive and explicit, and will try to balance editing in such points against undue tedium or tendentiously speculative length in the Discussion. In particular, we will note that a clear, simple implication of the work is to highlight an imperative to test CB839 in lupus patients already on hydroxychloroquine as standard-of-care, and to suggest development of UK5099 (already tested many times in mouse models of cancer) to complement glutaminase inhibition.

As backdrop, we note that the failure to advance imaging mass spectrometry to the capacity to quantify relative or absolute (via nano-DESI) concentrations of nutrients in localized interstitia is a critical gap in the entire field. Techniques that sample the interstitial fluid of tumour masses or in our case LN as a work-around have yielded evidence that there can be meaningful limitations of glucose and glutamine, but it needs to be acknowledged that such findings may be very model-specific and, as can be the case with cutting-edge science, are not without controversy. That said, yes, we had found that hypoxia reduced glutamine uptake but given the norms of focused, tidy packages only reported on leucine in an earlier paper [PMID27501247; PMCID5161594].

Beyond all that, another impetus to and inspiration for these experiments stems from quite data that we generated in a model of short-term protein-restricted diet (loosely akin to kwashiorkor in humans), based on an excellent publication showing that such a regimen quickly led to lower circulating glutamine and mTORC1 activity (**). In brief, we found that a low-protein diet did, in our experiments, preferentially lower glutamine but - importantly - led to reduced Ab responses (which would match what we have modeled here). The findings were not a well-enough connected evidentiary component to include in the "story" but I'll append slides with the relevant data to this Response to Reviews for the referee's perusal (and anyone else who reads this online discourse).

It would hence also be beneficial to test the CB839 + UK5099/HCQ combinations in a short, proof-of-concept treatment in vivo, e.g., shortly before and after the booster immunization or in an autoimmune model. Likewise, it may also be insightful to discuss potential effects of existing treatments (especially CB839, HCQ) on human memory B cell or PC pools.

We certainly agree that the suggestions offered in this comment are important next steps and the right approach to test if the findings reported here translate toward the treatment of autoimmune diseases that involve B cells, interferons, and pathophysiology mediated by auto-Ab. As practical points, performance and replication of such studies would take more time than the year allotted for return of a revised manuscript to eLife and in any case neither funds nor a lab remain to do these important studies.

Concrete evidence for our concurrence was embodied in a grant application to NIH that was essential for keeping a lab and doing any such studies. [We note, as a suggestion to others, that an essential component of such studies would be to test the effects of these compounds on B cells from patients and mice with autoimmunity]. Perhaps unfortunately for SLE patients, the review panelists did not agree about the importance of such studies. However, it can be hoped that the patent-holder of CB839 (and perhaps other companies developing glutaminase inhibitors) will see this peer-reviewed preprint and the public dialogue, and recognize how positive results might open a valuable contribution to mitigation of diseases such as SLE.

(2) Cell survival versus differentiation phenotype

Claims that the phenotypes (e.g., reduced PC numbers) are "independent of death" and are not merely the result of artificial cell stress would benefit from Annexin-V/active-caspase 3 analyses of GC B cells and plasmablasts. Please also show viability curves for inhibitor-treated cells.

This comment leads us to see that the wording on this point may have been overly terse in the interests of brevity, and thereby open to some odd misunderstanding. The CD138^+^ events are scored among VIABLE CELLS, so a decrease in the %CD138^+^ at similar division number represents an effect independent from (or beyond) survival and division-counting. Accordingly, we expanded the text of the Abstract and elsewhere in the manuscript, to be more clear. In addition, we added data from new experiments addressing death in vitro and among GC-phenotype B cells *in vivo*. To clarify in this public context, it is not that an increase in death (along with the reported decrease in cell cycling) can be or is excluded. The point is that beyond any such increase, and taking into account division number (since there is evidence that PC differentiation and output numbers involve a 'division-counting' mechanism), the frequencies of CD138^+^ cells and of ASCs among the viable cells are lower, as is the level of *Prdm1*-encoded mRNA even before the big increase in CD138^+^ cells in the population.

(3) Subset specificity of the metabolic phenotype

Could the metabolic differences, mitochondrial ROS, and membrane-potential changes shown for activated pan-B cells (Figure 5) also be demonstrated ex vivo for KO mouse-derived GC B cells and plasma cells? This would also be insightful to investigate following NP-immunization (e.g., NP+ GC B cells 10 days after NP-OVA immunization).

We performed a series of new experiments to have enough biologically independent replications for meaningful and statistical analyses. The new results, added in as Fig 5 - supplement 1, showed that the combined pathway interruption by loss-of-function increased ROS, mtROS, and death (annexin V / 7AAD) upon analyzing GCphenotype B cells immediately upon harvest. The findings align well with the data in Fig 5 (cultured B cells).

(4) Memory B cell gating strategy

I am not fully convinced that the memory-B-cell gate in Supplementary Figure 2d is appropriate. The legend implies the population is defined simply as CD19+GL7-CD38+ (or CD19+CD38++?), with no further restriction to NP-binding cells. Such a gate could also capture naïve or recently activated B cells. From the descriptions in the figure and the figure legend, it is hard to verify that the events plotted truly represent memory B cells. Please clarify the full gating hierarchy and, ideally, restrict the MBC gate to NP+CD19+GL7-CD38+ B cells (or add additional markers such as CD80 and CD273). Generally, the manuscript would benefit from a more transparent presentation of gating strategies.

In considering the referee's viewpoint, we further expanded the supplemental data displays to include more of the gating and analytic schemes, which we believe should mitigate one concern noted here. In addition, we now include flow data from the non-immunized control mice that had been analyzed concurrently in the experiments.

Third and finally, we performed new experiments and analyses in which the focus was the frequencies of memory-phenotype (IgD^neg^ GL7^neg^ CD38^+^ / CD38^hi^ aka CD38^++^) NPbinding B cells after immunization. While this time, as opposed to previously, the NP-APC staining met our standard for interpretability, the gist of the findings was that the two independent repeat experiments yielded a split decision and a degree of variability. With time being up due to the funds running out, we have elected to delete the issue and the data panel in question.

That said, it bears noting that in the previous figure panel, the labeling indicated that the gating included the important criterion that cells be IgD^neg^, which excludes the vast majority of naive B cells but measures memory-phenotype B cells independent from consideration of whether or not they were NP-binding.

In principle marginal zone (MZ) B cells might fall within this gate. However, the MZ B population is unlikely to explain the differences shown.

(5) Deletion efficiency - [The] mRNA data show residual GLS/MPC2 transcripts (Supplementary Figure 8). Please quantify deletion efficiency in GC B cells and plasmablasts.

Even were there resources to do this, the degree of reduction in target mRNA (*Gls*; *Mpc2*) renders this question superfluous. To the best of our understanding, the proteins (for which there might be some phenotypic lag) are translated from RNA. Might there be a small subpopulation of B cells (or their PC progeny) with only one, or even neither, allele converted from fl to D? Yes, but they would be a minor subset in light of the magnitude of mRNA reduction, in contrast to our published observations with *Slc2a1*. As to plasmablasts and plasma cells, the pre-existing populations make such an analysis misleading, while the scarcity of such cells recoverable with antigen capture techniques is so low as to make both RNA and genomic DNA analyses questionable. We also refer readers to the supplemental figure that presents the results of experiments testing the issue one might infer from the question about extents of deletion in PC (i.e., how much counter-selection might have occurred by the PC stage).
